# Identifying barriers to sustainable apple production: A stakeholder perspective

**DOI:** 10.1016/j.jenvman.2021.114082

**Published:** 2022-01-15

**Authors:** Shan Jin, Wenjing Li, Yiying Cao, Glyn Jones, Jing Chen, Zhenhong Li, Qian Chang, Guijun Yang, Lynn J. Frewer

**Affiliations:** a School of Natural and Environmental Sciences, Newcastle University, Newcastle upon Tyne, NE1 7RU, UK; b College of Economics and Management, Huazhong Agricultural University, Wuhan, 430070, China; c Institute for Agri-Food Research and Innovation, FERA Sciences Ltd., National Agri-Food Innovation Campus, Sand Hutton, York, YO41 1LZ, UK; d RSK ADAS Ltd, Spring Lodge, 172 Chester Road, Helsby, WA6 0AR, UK; e Institute of Agricultural Economics and Development, Chinese Academy of Agricultural Science, Beijing, 100081, China; f College of Geological Engineering and Geomatics, Chang'an University, Xi'an, 710054, China; g School of Engineering, Newcastle University, Newcastle upon Tyne, NE1 7RU, UK; h Key Laboratory of Quantitative Remote Sensing in Agriculture of Ministry of Agriculture and Rural Affairs, Beijing Research Center for Information Technology in Agriculture, Beijing, 100097, China; i Information Technology Research Center, Beijing Academy of Agriculture and Forestry Sciences, Beijing, 100097, China

**Keywords:** Apple production, Food system, Sustainability, Lock-in, Interpretive structural modelling, Policy

## Abstract

Apple is one of the most important cash crops in China. However, negative economic, environmental and social impacts are associated with its production. This study aims to apply a holistic systems perspective to understand existing problems associated with apple production in China and use this information to improve its sustainability. A structured survey was administered to farmers (n = 245) in Shandong and Shanxi provinces, combined with semi-structured interviews with apple supply chain stakeholders (n = 25). Themes, dimensions and relationships were identified based on an inductive thematic analysis of interview data, and then triangulated against the survey data. Interpretive Structural Modelling and Cross-Impact Matrix Multiplication Applied to Classification methods were applied to investigate interrelationships and effects of the elicited elements within the system. The results indicated that various environmental, economic and social problems are associated with apple production in China, including environmental and health risks associated with synthetic input applications, yield instability, deterioration of apple quality, farmers’ uncertainty about accessing routes to market, and the ageing farming workforce. The interaction of socio-economic and supply chain issues has contributed to the system “lock-in” to unsustainable practices within the apple production system. Existing agricultural policies were ineffective as they did not include policy leverage to mitigate the multiple factors driving lock-in to unsustainable practices within the system. The research has provided evidence to enable policymakers to develop effective and targeted strategies to facilitate sustainable production within the apple production system. In particular, the future policy mix should consider the entirety of the food system including perspectives and requirements of different stakeholders. The three-stage approach applied has demonstrated its feasibility of investigating sustainability issues facing a particular industry within a specific cultural and policy context.

## Highlights

• 21 barriers to Chinese sustainable apple production were identified.
• Vicious circles trap different problems in the apple production system.
• There exists socio-technical lock-in around existing tools and practices.
• Ineffective agricultural policies strongly hinder upgrading apple production.
• A three-stage approach was established for researching agricultural sustainability.

## Graphical abstract

**Figure:**
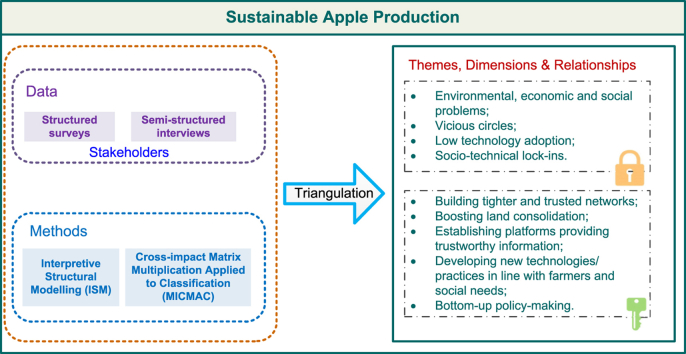

## 1 Introduction

Promoting sustainable agriculture and, at the same time, delivering food security represents a challenge for many countries (UN, 2015). Global increases in demand for foods have resulted in the use of more synthetic agricultural chemicals and natural resources (e.g. land and freshwater) for food production, which has the potential for negative impacts on the environment and public health (Godfray et al., 2010). Many people who are engaged throughout the entirety of the supply are dependent on agriculture for their livelihoods. It is important to simultaneously consider environmental, economic, and social issues associated with food production, to ensure agrifood systems are “environmentally non-degrading, technically appropriate, economically viable, and socially acceptable”, thereby facilitating their transition to a higher level of sustainability (FAO, 1989, p. 65; Pretty, 2008).

A variety of environmental, economic, and social problems have been identified within agricultural systems. These include environmental pollution associated with primary production, negative environmental and health impacts of food processing, and supply chain logistics, negative impacts on natural resource conservation, for example, water and biodiversity, high levels of energy consumption; volatile pricing of agricultural products, unstable farmer access to markets, shortages of farm labour, low farm incomes and farmer dependence on subsidies; negative impacts on food availability and safety, and on the well-being of farming communities and society (e.g. see Maye et al., 2018; Meuwissen et al., 2019; Van Cauwenbergh et al., 2007; Zhu et al., 2018).

Farmers' adoption of new practices and technologies may represent an effective strategy to address some of these problems (Pretty et al., 2018). Examples include the adoption of integrated pest management (IPM), which reduces pesticide application (FAO, n.d.), or increased mechanization which improves the economic prospects of a farm by reducing labour inputs (Clark et al., 2018). While technological and practical innovations potentially bring various environmental, economic and social benefits, diffusion to farmers and other end-users may be slow (Yigezu et al., 2018), representing a barrier to achieving sustainable production. The underlying factors driving slow innovation diffusion are embedded within social networks, and include heterogeneous characteristics of farmers and farm families, agronomic conditions, socio-economic factors and the policy context in which farmers make decisions (Dessart et al., 2019; Li et al., 2020; Montalvo, 2008; Pannell et al., 2006; Yigezu et al., 2018). Relevant characteristics of farmers which facilitate, or act as barriers to, the adoption of sustainable agronomic practices could include, *inter alia*, benefit and risk perceptions associated with specific problems or technologies, personal risk attitudes linked to adoption of innovations, and socioeconomic and demographic status (Li et al., 2020; Yigezu et al., 2018). For example, the evidence has indicated Chinese farmers with higher dependence on farming as a source of income are more likely to adopt precision agriculture equipment (Li et al., 2020). Farm attributes (such as the size, degree of land fragmentation, location, and access to irrigation water) and technology/practice attributes (such as the extent to which the functions of technologies are in line with farmers' needs) may also need to be considered in novel technology/practice promotion (Clark et al., 2018; D'Emden et al., 2008).

The literature on agricultural sustainability depicts a complex and dynamic agricultural system, where multiple problems can potentially emerge, and where interrelations exist within and between different types of agricultural problems and their drivers. For example, production-related pollution could pose health risks to farmers, and recreational users and rural residents, as well as consumers of contaminated food products and the environment (Cole et al., 2011). Income instability and labour shortages on farms may exacerbate rural-urban drift, compromising farmland succession, which has been identified as a problem in different countries including Ireland, China and the United Kingdom (e.g. see Fischer and Burton, 2014; Hennessy and Rehman, 2007; Zou et al., 2018). Adoption of new practices and technologies may result in unintended or new agronomic and social problems, or indeed the reshaping of the agronomic system under consideration (Knowler and Bradshaw, 2007).

As part of this, there is a need to understand why farmers maintain the use of existing tools and practices, as well as why they do not adopt new ones. David (1985) has argued that “socio-technical lock-in” might occur when a particular technology or practice (described as “incumbent”) has become normative or standard within a specific area of application. This is partly because the incumbent technology or practice benefits from the “increasing returns of adoption”. As more people adopt a particular technology or practice, performance may improve through economies of scale, end-user familiarity, adaptive expectations and network effects (e.g. established networks for accessing needed information), subsequently leading to further adoption. Subsequently, other compatible technologies and practices will be developed to reinforce the dominant position of the incumbent ones (Arthur, 1989). It is important to assess whether farmers are locked-in to incumbent technologies and practices, what factors reinforce lock-ins, and how the impacts of locked-in situations can be reduced to ensure the adoption of environmentally beneficial, but innovative, technologies and practices.

Cases of locked-in situations within the agricultural sector, in relation to certain incumbent technologies and practices that are habitual to farmers and hard to change, have been infrequently investigated. Examples include the continued use of synthetic pesticides despite the availability of more sustainable alternatives (Cowan and Gunby, 1996; Flor et al., 2019, 2020; Wagner et al., 2016), or the continued cultivation of traditionally planted plant cultivars despite the availability of new cultivars with more benefits to the environment and society (Magrini et al., 2016; Meynard et al., 2018; Vanloqueren and Baret, 2008). The locked-in concept can also be identified concerning farmers’ reversion to habitual practices/technologies after a short-period adoption of agricultural innovations, potentially because of failure to address the needs of a broad range of stakeholders within a supply chain (Magrini et al., 2016). Failure to provide social and economic support to innovation may also have negative impacts on its adoption. For example, the adoption of precision agriculture technologies among Chinese farmers decreased from 53.2% to 12.0% due to lack of farmer access to technical service providers and financial limitations (Li et al., 2020).

This research aims to use a holistic approach to understand how different factors shape the sustainability of the Chinese agricultural production system. China's apple production is important to consider from the perspective of agricultural sustainability for several reasons. First, apple is one of China's most important cash crops. In 2018, China was ranked 1st in the world in terms of apple production volume. However, its production volume per hectare was ranked 32nd, indicating a relatively low production efficiency (FAO, 2019). As apple is primarily produced by smallholder owned farms, higher production efficiency is required to increase smallholder farmers' incomes (Wang et al., 2016). Second, despite China being responsible for 46% of world apple production, its apple exports accounted for 14% of the global trade as international food safety standards are not met, for example in relation to excessive pesticide residues (FAO, 2019; Snyder and Ni, 2017). Significant environmental pollution in China has been attributed to excessive use of fertilizers and pesticides in agricultural production, threatening public and environmental health (Zhao et al., 2017). There is therefore a need to *simultaneously* address problems associated with production efficiency, food safety and environmental considerations linked to apple production. Third, apple production tends to result in more negative environmental impacts when compared to, for example, traditional crop farming (Pang et al., 2019; Wagner et al., 2016). However, understanding the determinants of sustainable farming practices associated with China's apple production systems has not been a focus of research. Fourth, despite more sustainable practices and technologies being developed in China and potentially applied to apple production (Yang et al., 2017, 2019), their adoption by apple producers has infrequently been investigated.

This study seeks to address the following research questions:

• What are the key environmental, economic, and social problems within China's apple production systems?
• Have innovative technologies and practices effectively addressed these problems? Why or why not?
• How do various elements (i.e. key factors either shaping, or hindering transformation of, the current sustainability status of apple production) interact with each other within the apple production system?
• What are the implications for improving the sustainability of China's apple production?

## 2 Methodology

Combining quantitative and qualitative data enables a more detailed and balanced understanding of an agricultural system. This type of triangulation has been applied in social science research as a way of ensuring reliability and validity (Lewis-Beck et al., 2004). Here, a mixed methodology combing the results of a structured survey and semi-structured interviews was used to explore environmental, economic, and social problems, and their interactions, in China's current apple production system. Such a mixed methodology has been used to study agriculture-related issues, such as the development of Brazilian commodity agriculture (Hajjar et al., 2019), and Danish farmers' adoption of precision farming (Pedersen et al., 2004). Ethical approvals for the survey and interviews in this study were granted by the lead researcher's university (Ref: 18226/2019), and data collection was conducted in September 2019.

### 2.1 Structured survey

A questionnaire informed by the existing literature on agricultural sustainability was developed (e.g. see Fan et al., 2015; Hao et al., 2018; Ma, Abdulai and Goetz, 2018; Zhang et al., 2019). Questions were adapted to make them specific to the context of apple production, and primarily focused on farmers’ current orchard management practices (e.g. their use of pesticides, fertilizers and water in irrigation), apple sales, and technology (e.g. integrated water and fertilizer equipment) and service adoption (e.g. agricultural insurance and participation in apple cooperatives), as well as access to sales and participation in training courses about apple cultivation. The survey was developed in Chinese and piloted with five apple farmers on July 31 and August 1, 2019.

**Fig. 1:**
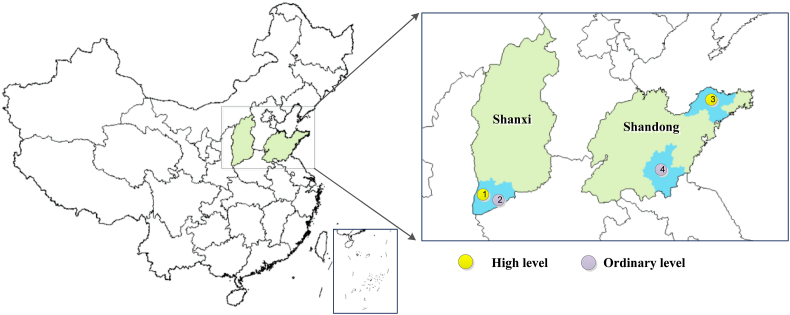
Sampling areas for the survey. The yellow dots indicate sampling areas with high-level apple production and the purple dots with the ordinary-level production. (For interpretation of the references to colour in this figure legend, the reader is referred to the Web version of this article.)

Shaanxi and Shandong provinces are the two most important apple production regions, whose production volume accounted for 25.7% and 24.3% of the national apple yield in 2018 (National Bureau of Statistics, 2019). There are six other main apple production regions, including Henan (10.3%), Shanxi (9.6%), Gansu (7.4%), Liaoning (6.0%), Hebei (5.6%) and Xinjiang (4.2%). To ensure the representativeness of our selected provinces as well as the data quality with a limited budget, we decided to focus on one more and one less developed region and use face-to-face surveys for data collection. Therefore, we finally selected Shandong and Shanxi provinces as our focused apple production regions. To further reduce selection bias, two counties were chosen from each province based on a combination of apple production volume and quality, representing a relatively high-level (Linyi for Shanxi and Qixia for Shandong) and low-level (Yanhu for Shanxi and Yishui for Shandong) of production in relation to these two characteristics across provinces (Fig. 1). Farmers from these counties were then randomly selected for surveys, conducted by trained enumerators.

The questionnaires were distributed to 253 apple farmers in Shandong and Shanxi provinces. Of these, a total of 245 questionnaires were retained for further analysis after the removal of those with missing data. The average age of respondents was 55, with a mean apple cultivation experience of 21 years. 82% of these respondents had less than 12 years of education, and male farmers accounted for 87% of the sample. In terms of apple production, the Fuji apple is the most cultivated variety in the two provinces (96%). The respondents’ mean orchard size is 0.76 ha, and the income from apple orchards contributes an average of 72% to their household income (Table 1).

**Table 1:** Survey sample demographics.

Variable	Category	Total		Shandong		Shanxi	
		n	Freq.	N	Freq.	n	Freq.
**Gender**	Male	214	87%	101	85%	113	90%
	Female	31	13%	18	15%	13	10%
**Age**		***54.90***		***55.19***		***54.64***	
	Under 50	60	25%	25	21%	35	28%
	50–59	113	46%	62	52%	51	40%
	60 and above	72	29%	32	27%	40	32%
**Education**		***8.31***		***7.30***		***9.25***	
	<6 years	28	12%	27	23%	1	1%
	6–8 years	54	22%	29	24%	25	20%
	9–12 years	118	48%	49	41%	69	55%
	12 years and above	45	18%	14	12%	31	24%
**Farming years**		***21.28***		***20.13***		***22.37***	
	Less than 20 years	131	53%	65	55%	66	52%
	20 years and above	114	47%	54	45%	60	48%
**Orchard size**		***0.76***		***0.57***		***0.94***	
	less than 1 ha	187	76%	108	90%	79	63%
	1 ha and above	58	24%	11	10%	47	37%
**Number of blocks**		***3.06***		***3.13***		***2.98***	
	1	63	26%	35	29%	28	22%
	2	56	23%	25	21%	31	25%
	3 and above	126	51%	59	50%	67	53%
**Apple income ratio**		***71.78***		***71.82***		***71.75***	
	Less than 50%	74	30%	35	29%	39	31%
	50% and above	171	70%	84	71%	87	69%

Note: Figures in Italic refers to the mean of each variable.

### 2.2 Semi-structured interviews

Semi-structured interviews are an effective method for exploring respondents' views and collating in-depth information about complex issues (Barriball and While, 1994). This method has been widely used to investigate farmers' and other stakeholders’ attitudes and decision-making associated with agricultural issues (e.g. see Lamprinopoulou et al., 2014; Nguyen et al., 2016; Oliveira et al., 2013). Supply chain-oriented and other broader stakeholder categories based on apple supply chain activities were selected, and semi-structured interviews with representatives of these stakeholders were conducted, in order to identify a range of perspectives about the factors which underly the sustainability within the entire apple supply chain.

A total of 25 participants who represented different apple supply chain stakeholders' views were recruited for interviews, with some engaging in multiple roles within the supply chain. Interviewees' identities were anonymized other than stakeholder categorization, within (e.g. production, warehousing, sales, purchase and logistics) and linked to (e.g. policy implementation and research into production and markets) the apple supply chain (see Appendix A Table S1). The interview questions investigated interviewees’ views on the current apple production in China and their views on associated environmental, economic and social problems. The number of interviews was determined according to the requirement for data saturation, when no more new themes were identified from the last interview (Guest et al., 2020). All the interviews were digitally recorded and transcribed *verbatim* in Chinese.

### 2.3 Data analysis

An inductive thematic analysis was applied to the interview data, through which themes, dimensions and relationships emerged from the data without being determined *a priori*, utilising data familiarization; code generation; and construction, revision and definition of themes, and report production (Braun and Clarke, 2006). The coding process was facilitated by QSR International's NVivo 11 software. The inductive approach has been used in China to explore farmers' decision-making about water-saving irrigation (Burnham et al., 2015), responses to climatic variability and land-use change (Hageback et al., 2005), and perceptions of drought policy implementation (Pradhan et al., 2017). The survey represented a baseline investigation to identify topics for in-depth analysis in the subsequent interviews. The results were triangulated against the interview data to reduce the bias associated with the small interview sample size.

In order to identify interactions between the elicited themes and dimensions that emerged from the thematic analysis, Interpretive Structural Modelling (ISM) was employed, which offers a qualitative modelling language to structure directly and indirectly related elements, and thus builds a comprehensive model to enable understanding for a complex phenomenon or system (Janes, 1988; Malone, 1975). Based on the relationships of different elements (i.e. themes identified *via* thematic analysis), the followed process included developing Reachability Matrix and modifying the matrix by considering transitivity; levels partitioning of elements and drawing the hierarchical structural model; and checking conceptual consistency and making necessary changes to the model (Agi and Nishant, 2017; Ahmad et al., 2020; Raci and Shankar, 2005). Details of ISM as applied here are presented in Appendix B. To help understand the role of each element in shaping a phenomenon or system, the Cross-Impact Matrix Multiplication Applied to Classification (MICMAC) method was employed to categorize elements within the system into autonomous, dependent, linkages and driving elements, according to their driving and dependence power (Kanungo et al., 1999). The integration of ISM and MICMAC methods has been used to explore interactions of elements in relation to sustainability issues (e.g. food wastage within perishable food supply chains, and adoption of environmentally friendly practices in different industries) (e.g. see Agi and Nishant, 2017; Balaji and Arshinder, 2016; Gardas et al., 2019b; Ravi, 2015; Ren et al., 2015), but not, to our knowledge, in relation to sustainable apple production.

## 3 Results

### 3.1 Problems faced by apple production in China

Key environmental, economic and social problems in China's apple production were identified through the semi-structured interviews and the surveys. These problems were sometimes described as being difficult to address due to multiple underlying drivers and complex interactions within and between problems and drivers. The problems were categorized into four major themes, including environmental and health risks, yield instability, deterioration of apple quality and farmers' uncertainty about accessing routes to market (details see Appendix A Table S2).

#### 3.1.1 Environmental and health risks

**Fig. 2:**
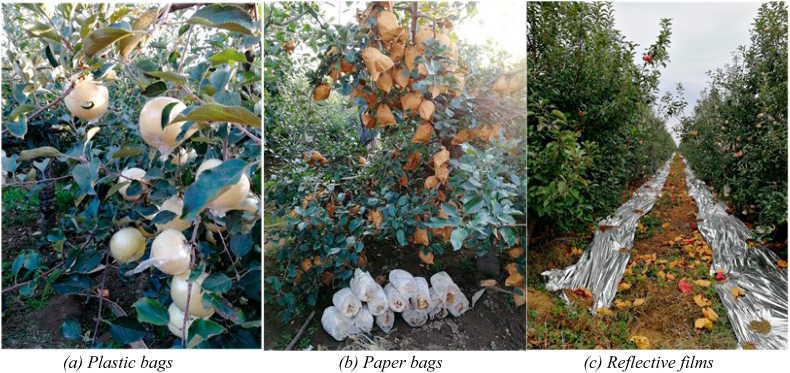
Apple bagging and reflective films in apple orchards from field work.

Synthetic agricultural chemicals were a source of environmental contamination in apple production (Evidence see 3.1.1.1 in Table S2). Most farmers did not realise their lack of knowledge about orchard management, resulting in environmental problems linked to the improper use of synthetic fertilizers and pesticides. Spraying chemicals, negative health impacts might be experienced by bystanders and residents, despite farmers’ use of personal protective equipment (Evidence see 3.1.1.2 in Table S2). In addition, apple bagging was used for protecting apples from certain pests, promoting apple skin colouration and reducing blemishes. In the absence of effective disposal and recycling mechanisms, the plastic bags, together with reflective films in orchards, represented a new environmental threat (see Fig. 2) (Evidence see 3.1.1.3 in Table S2).

#### 3.1.2 Yield instability

Yield instability was a serious problem for farmers. Extreme climate events were considered to cause apple yields to plummet in certain areas, triggering price spikes across the country, demonstrating that the production system had low resilience to climate shocks. The changes in apple supply and demand then led to the instability of apple prices. While some stakeholders mentioned the malicious hoarding of apples by big investors, the futures company manager suggested that this only exerted a very small influence on national price volatility (Evidence see 3.1.2.1 in Table S2). Cultivating dwarf varieties was considered to be able to mitigate negative impacts of climate conditions on production. However, it can be a heavy financial burden for apple producers, in particular smallholder farmers, given the extra expenditure on saplings and supporting facilities and the income loss due to about 3 years’ growth to maturity (Evidence see 3.1.2.2 in Table S2). Despite the potential of agricultural insurance in mitigating financial risks caused by climatic extremes, farmers expressed low trust in insurance companies, resulting in the failure in promoting the insurance (Evidence see 3.1.2.3 in Table S2).

#### 3.1.3 Potential deterioration of apple quality

Bigger, redder and blemish-free apples were often regarded as being of higher quality and could be sold at a higher price. Given the potential negative influence of pests, plant disease and climatic extremes, farmers were motivated to use farm chemicals, bagging and reflective films to ensure the “visual” quality of apples. The taste of apples appeared to have been negatively affected by the misuse of apple bagging and synthetic fertilizers. However, farmers often failed to realise their overuse of farm chemicals (Evidence see 3.1.3.1 in Table S2).

#### 3.1.4 Farmers’ uncertainty about accessing routes to market

Smallholders mainly depended on small independent brokers, local apple companies or warehousing companies for sales, and normally had no regular contact or formal collaborations with wholesalers and retailers. In contrast, larger-scale farmers and apple companies tended to have more stable collaborations with wholesalers and retailers (Evidence see 3.1.4.1 in Table S2). Farmers’ limited market access, when combined with their weak bargaining power relative to that of wholesalers and retailers, has led to uncertainty about how to initiate apple sales, causing negative impacts in particular on smallholders. In addition, there has been lack of a trusted supplier-buyer relationship (between farmers and buyers) as the fruit supply chain companies, the wholesalers and the retailers primarily collaborated with apple companies or agricultural cooperatives (Evidence see3.1.4.2 in Table S2).

### 3.2 Limited effect of promoted technologies and practices

Farmers frequently expressed an unwillingness to adopt innovative technologies and/or practices. This included, for example, the planting of dwarf apple trees, purchasing new equipment for improved irrigation and weeding, and purchasing services from the soil testing and fertilizer recommendation service. Various factors that led to farmers’ low adoption of new technologies and practices were identified as follows.

#### 3.2.1 Land fragmentation and low standardization between orchards

Land fragmentation limited farmers' adoption of mechanization (e.g. irrigation and weeding), as it required sufficient orchard size and tree spacing. It was difficult to persuade farmers to standardize tree spacing as it potentially resulted in financial losses by reducing the number of trees planted. Consequentially, orchard management has become inefficient due to low standardization and mechanization, which had negative effects on apple yields and farmers’ income (Evidence see 3.2.1.1 in Table S4).

#### 3.2.2 Lack of long-term apple cultivation plans

The ageing farming workforce and young people's rural-to-urban migration was a frequently mentioned problem and acted as a barrier to the long-term plan for improving apple production. Despite the government's offer of training courses for young people, few ultimately continued working in apple production (Evidence see 3.2.2.1 in Table S4).

#### 3.2.3 Financial difficulties

Farmers had limited budgets for investment in technologies. As such, farmers’ access to financial support might significantly affect their investment in apple production. For those who planned to expand their orchard sizes, the difficulty of getting loans from financial institutions could act as a barrier to shift to more environmentally friendly technologies and practices (Evidence see 3.2.3.1 in Table S4).

#### 3.2.4 Low awareness and interests of sustainable orchard management

Overall, farmers had low awareness of and interests in sustainability issues associated with apple production. Other stakeholders such as the wholesaler and retailer mentioned the importance of environmental conservation. However, they did not include the environmental impacts of production as a criterion for supplier selection, representing lack of buyer pressure to drive sustainable production (Evidence see 3.2.4.1 in Table S4).

#### 3.2.5 Limited access to trustworthy information

Training is an effective way for farmers to improve their knowledge and skills in sustainable farming practices, but some training farmers attended was provided by chemical manufacturers (Evidence see in 3.2.5.1 Table S4). Also, no regular contact had been established between farmers and big retailers or wholesalers. Farmers depended on small independent brokers, apple companies or warehousing companies for sales, but with a low trust, which could act as a barrier to information exchange, for example, about wholesalers' or retailers’ procurement (Evidence see 3.2.5.2 in Table S4).

#### 3.2.6 Limited development and promotion of novel technologies and practices

Difficulties and failures associated with novel technology/practice promotion in apple production areas reflected low adoption willingness among farmers. A lack of technologies that help with the proper use of pesticides in apple cultivation was identified (Evidence see 3.2.6.1 in Table S4).

#### 3.2.7 High levels of risk perceptions linked to financial loss

Farmers were concerned about yield loss and quality decline due to the significant impact on income. Therefore, they actively used chemicals to protect apple trees from pests and diseases, a behaviour which could be difficult to change. Farmers might perceive that reducing chemical inputs increases the risk of yield loss or has negative effects on the visual quality of the apples (Evidence see 3.2.7.1 in Table S4).

#### 3.2.8 Ineffective agricultural policies

The Chinese government provided subsidies for purchasing organic fertilizers, but farmers were still more dependent on synthetic fertilizers. Existing agricultural policies did not include some potentially problematic areas of production. For example, policies targeting the reduction of pesticide use and other environmental pollutants (e.g. plastic bags) have not been enacted (Evidence see 3.2.8.1 in Table S4). Many agricultural cooperatives were established in different apple production regions after the Law of the People's Republic of China on Farmers' Professional Cooperatives was implemented. They were intended to benefit smallholder farmers by providing different services, such as technical training, facilitating apple sales and enabling collective purchases of agricultural inputs. In fact, a few cooperatives in Shanxi province were founded by apple companies, pesticide or fertilizer retailers or warehousing companies. Some interview participants questioned the value of the functions or services agricultural cooperatives provided for farmers and considered these cooperatives primarily as a means of obtaining financial incentives from the government (Evidence see 3.2.8.2 in Table S4).

### 3.3 System elements interact and exhibit different influences on the system

The findings in Section 3.1, 3.2 included 21 elements: E1- Environmental and health risks, E2-Yield instability, E3-Deterioration of apple quality, E4-Farmers’ uncertainty about accessing routes to market, E5-The improper use of agricultural chemicals, E6-The use and disposal of plastic bags and reflective films, E7-Lack of orchard management knowledge among farmers, E8-Effects of extreme weather and climate change, E9-Lack of buyer pressure to drive sustainable production, E10-Weak farmer bargaining power, E11-Lack of trusted supplier-buyer relationship, E12-Land fragmentation and low standardization between orchards, E13-Farmers’ lack of long-term apple cultivation plans, E14-The ageing farming workforce and young people's rural-to-urban migration, E15-Farmers’ financial difficulties, E16-Low farmer awareness of, and interest in, sustainable orchard management, E17-Limited access to trustworthy information about apple production and sales, E18-Limited development and promotion of novel technologies/practices, E19-High risk perceptions regarding potential financial losses, E20-Low farmer willingness to adopt novel technologies/practices, E21-Ineffective agricultural policies (see bold phrases in Appendix A Table S2 and S4).

#### 3.3.1 Interactions of system elements

**Fig. 3:**
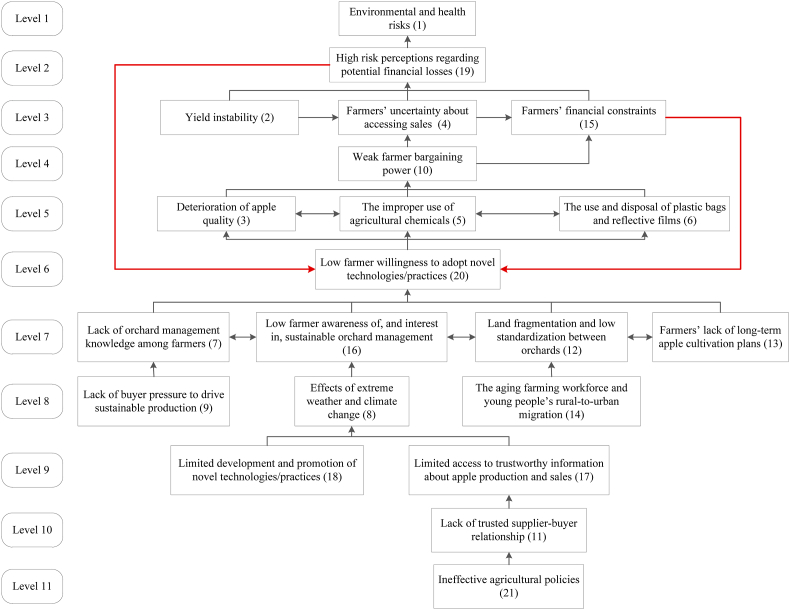
Hierarchical structural model of key elements in Chinese apple production.

The identified elements appear to interact with each other and are a part of a dynamic and complex system. Applying ISM methodology (analysis details see Appendix B) has enabled the system elements identified to be placed in a hierarchical structure (Fig. 3). The key elements identified in Chinese apple production, including environmental and health risks (E1), yield instability (E2), deterioration of apple quality (E3) and farmers' uncertainty about accessing routes to market (E4), are placed at upper levels within the hierarchy (level 1 to 5), and are linked to farmers having high levels of risk perception associated with potential financial losses (E19). These are directly caused by yield instability (E2) and/or uncertainty about accessing routes to market (E4) and/or financial difficulties (E15), which could amplify the environmental and health risks in apple production (E1). In other words, environmental and health risks can be mitigated only if actions have been taken to address the other problems, which will reduce farmers' high levels of concerns linked to financial losses (E19). Farmers’ weak bargaining power in transactions (E10 at level 4) indirectly increases their risk perceptions linked to financial losses during apple cultivation by increasing the level of uncertainty in sales and the resulting financial difficulties. Meanwhile, E10 can be directly affected by the deterioration of apple quality, and indirectly by the improper use of agricultural chemicals and plastic bags.

Fig. 3 shows two loops (E19 and E20, E5 and E20) between level 1 and 6, where a series of problems either worsen farmers' financial difficulties or increase their risk perceptions associated with potential financial losses, and in turn negatively affect their adoption of new technologies and practices, thereby reinforcing the negative feedback loops. The other elements that directly reduce farmers' adoption of new technologies and practices include lack of orchard management knowledge, low awareness of, and interest in, sustainable orchard management, and lack of long-term cultivation plans, and land fragmentation and low standardization between orchards at level 7; lack of buyer pressure to drive sustainable production at level 8; limited development and promotion of novel technologies/practices, and farmers' limited access to trustworthy information about production and sales at level 9; and ineffective agricultural policies at level 11. The ageing farming workforce and young people's rural-to-urban migration (level 8), and lack of trusted supplier-buyer relationship (level 11) can also indirectly result in farmers' low willingness to adopt new technologies and practices. An interesting finding relates to the effects of extreme weather and climate change (E8 at level 8), which causes a few problems in apple production (e.g. yield instability and deterioration of apple quality), while potentially increasing farmers' adoption of new technologies (E20 at level 6) in particular those mitigating climate risks to production by raising farmers' awareness of the need to upgrade orchard management (E16 at level 7).

#### 3.3.2 Role of key elements in shaping the system

**Fig. 4:**
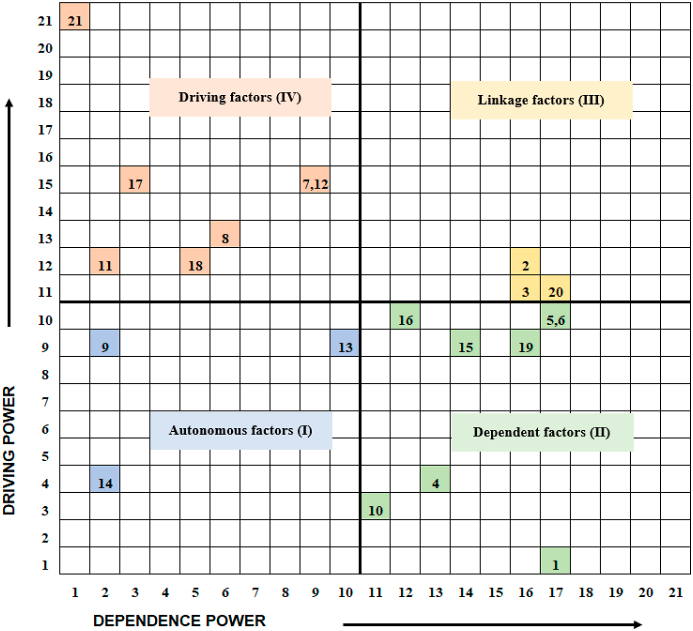
MICMAC analysis of elements shaping the sustainability status of apple production.

MICMAC analysis categorized the 21 key elements into four clusters based on their driving and dependence power, which has enabled insights into effects of these elements within the system to be identified (Fig. 4). Autonomous elements are located in Quadrant I. These elements have weak driving and dependence power and are relatively disconnected from the system. They often have few links, while these links could be strong. 3 of the 21 identified elements including E9, E13 and E14 were assigned to this category. Dependent elements are located in Quadrant II. They simultaneously have weak driving power and strong dependence powers and are less capable of influencing others. Elements falling within this category in the model include E1, E4, E5, E6, E10, E15, E16 and E19. Linkage elements have both strong driving and dependence power and are placed in Quadrant III. As the effect of other elements passes through them, linkage elements can either amplify or weaken the effect of others. Elements pertaining to this category are E2, E3 and E20. Driving elements (also named as “independent” elements) have strong driving power but weak dependence power and are located in Quadrant IV. These elements exert effects on most of the other elements and are therefore essential for understanding the behaviour of this system. This category contains E7, E8, E11, E12, E17, E18 and E21, which shape the status of the system but are not or minimally influenced by the other system elements. Changes linked to these elements should be prioritized to address so as to improve the sustainable development of apple production in China.

## 4 Discussion

The results of this research have enabled the identification of various environmental, economic and social issues Chinese apple production has been facing, including environmental and health risks, yield instability, deterioration of apple quality and farmers' uncertainty about accessing routes to market (see also Table S2. Key environmental, economic and social problems in China's apple production). There is also evidence presented to suggest that these issues are potentially interactive. While new practices and technologies have the potential to address some of these problems, their promotion has been hindered due to multiple factors embedded in the agronomic, social and economic context of apple production systems. For example, farmers' low intention to adopt new more efficient production practices is driven by economic factors on farms, low awareness of, and interest in, sustainable orchard management, and ineffective existing agricultural policies which do not address the system as a whole. In particular, based on the interactions of these identified system elements, the potential of “vicious circles” or negative feedback loops to “trap” the problems in the system was identified, which could reinforce the unsustainably of apple production.

### 4.1 Lock-in around synthetic pesticides

The lock-in around synthetic pesticides in Chinese apple production has posed a high risk to the environment. In China, the number of pesticide manufacturers, and investment in research and development associated with synthetic pesticides, has increased rapidly in the past four decades (Wu et al., 2018). This has defined the use of synthetic pesticides as a standard for pest control. The low price of pesticides, farmers’ accumulated knowledge about pesticides, lack of access to information about sustainable production, prior positive experience of synthetic pesticide use, and reliance on information and training provided by pesticide manufacturers could reinforce the lock-in within the apple production system, preventing farmer adoption of alternative strategies (Arthur, 1989; Cowan and Gunby, 1996). The practice of apple bagging could even reinforce pesticide lock-in in apple production. While bagging could theoretically decrease dependence on pesticides in fruit production (Polidoro et al., 2008; Sharma et al., 2014), farmers had not reduced pesticide applications because farmers perceived that the bags reduced apple pesticide residues following application.

Innovation niches could develop alongside dominant technologies, which may contribute to the unlocking of the dominant system if other social and economic conditions are favourable (Geels, 2002). Collet and Mormont (2003) suggested dismantling pesticide lock-in in apple production *via* better coordination between actors and pesticide-related information sharing within the supply chain. The results of our study indicate that a few Chinese apple farmers have adopted mechanical and/or biological methods for pest control, (see also Ma and Abdulai, 2019). Chinese consumers, in particular younger people, have expressed preferences and willingness to pay price premiums for food with reduced chemical residues (Yu et al., 2014), and so, a niche market for more sustainably produced apples within China can be identified to encourage farmer adoption of more sustainable practices.

### 4.2 Low adoption intention of new technologies and practices

Apple farmers in this study expressed low willingness to adopt new technologies and practices, including those potentially benefiting the environment, which was affected directly by E7, E9, E12, E13, E15-E19 and E21, and indirectly by E2, E3, E4, E8, E11 and E14. E2, E3, E4, E15, E16 and E19 were assigned to either dependent or linkage elements, which could be largely driven by the other elements within the system (see Fig. 4). The remaining direct E7, E9, E12, E13, E17, E18 and E21, and indirect E8, E11 and E14 should be considered as potential routes first to facilitate farmers’ willingness to adopt new technologies and practices.

Farmers’ knowledge and access to information about farming (E7 and E17) played an important role in their decision making about new technologies and practices. More training courses, for example, in relation to sustainable orchard management practices, should be provided to farmers (Zhao et al., 2008). Using Information and Communication Technologies (ICT) has been found to improve agriculture information dissemination and knowledge transfer in China (Zhang et al., 2016). However, Chinese apple farmers found it difficult to evaluate the reliability of production information on the internet. Also, some training courses for farmers were provided by fertilizer or pesticide manufacturers, who were unlikely to encourage farmers to reduce the use of agricultural chemicals. Therefore, trustworthy platforms for publishing information about environmentally friendly technologies and practices for orchard management should be collectively co-produced by stakeholders to help smallholder apple producers to develop sustainable apple farming practices.

In line with previous research (Kumar and Rahman, 2016; Seuring and Müller, 2008), the importance of farmers' social networks in affecting their adoption of sustainable orchard management technologies and practices has been demonstrated (E9 and E11). In the absence of stable relationships between farmers and buyers (e.g. wholesalers and retailers) within the apple supply chain, the exchange of information about production and sales has been limited, including in relation to sustainable orchard management. Buyers have been shown to exert significant influence on suppliers' adoption of sustainability practices through integrating environmental and social issues into supplier selection, for example, requiring products to meet certain standards (e.g. ISO 14001) (Beske et al., 2008; Kumar and Rahman, 2015). However, big wholesalers and retailers in China tend to collaborate with apple companies or agricultural cooperatives rather than smallholder farmers, which destabilizes the potential for supplier-buyer relationships between farmers, and wholesalers and retailers (E11). This may weaken the impacts of buyer pressure to drive sustainable primary production, given that smallholders represent the majority of apple producers in China (Wang et al., 2016). Also, big wholesalers and retailers are not interested in including environmental considerations as a factor determining their supplier selection. Even if buyers and retailers intend to address sustainability issues within the supply chain, monitoring suppliers’ sustainability performance may be problematic unless effective traceability systems (e.g. using blockchain technology) are established for recording sustainability information through the entirety of the supply chain (Kamilaris et al., 2019).

Climate extremes have been considered to negatively influence apple yield and quality (Dalhaus et al., 2020), which was also identified as a stakeholder concern in this study (i.e. E8). Fig. 3 shows that the negative impacts of extreme weather could be amplified by limited access to trustworthy information about apple production and sales (E17) and limited development and promotion of novel technologies/practices (E 18). Research is needed to ensure the development and promotion of novel environmentally friendly technologies and practices aligns with farmers' (perceived) needs. Climate extremes have acted to raise farmers’ awareness about climate risks, while most smallholders have yet to actively adopt adaptation strategies (e.g. planting dwarf apple trees), due to financial considerations.

Land fragmentation has been one of the main problems facing Chinese agriculture and has negatively influenced apple farmers' adoption of novel technologies (E12). Tan et al. (2008) have reported negative impacts of land fragmentation on Chinese rice farmers' technology adoption, and have suggested the implementation of land consolidation programs to remedy this. While land consolidation has been implemented *via* land transfers in the areas included in this research, land fragmentation still existed. The ageing farming workforce and young people's rural-to-urban migration in apple production (E14), and the resulting farmers' lack of long-term apple cultivation plans (E13) were identified, which appeared to further reduce farmers' adoption of technology. For a range of different countries, the existence of fewer young farmers might lead to a loss of potential in establishing more profitable and sustainable agricultural production, assuming young farmers tend to be more innovative, entrepreneurial and amenable to change (Hamilton et al., 2015; Zagata and Sutherland, 2015). Younger farmers also tended to be more willing to invest and engage in agri-environmental schemes, thereby playing an important role in addressing challenges posed by climate change (Davis et al., 2013; Sutherland et al., 2016).

### 4.3 Limited role of agricultural cooperatives

The Chinese government has promoted the establishment of agricultural cooperatives in apple production regions, which could solve some problems in the negative feedback loops by increasing farmers' bargaining power and income *via* collective actions (Bijman and Iliopoulos, 2014). The positive impact of cooperative membership on apple farmers' household welfare (e.g. increased technical efficiencies, apple yields, net returns and household income) and on investment in organic soil amendments in China's apple production has been demonstrated in previous research. The researchers, therefore, suggested that the government should intensify support for, and encourage farmers to join, cooperatives if sustainable production sustainably is to be increased (Ma et al., 2018a, 2018b; Ma and Abdulai, 2016). However, the six selected cooperatives were specialized in apple production and marketing, and so not representative of all cooperatives. In addition, the results suggested that some cooperatives have limited functions, and are sometimes considered only as a mechanism for obtaining financial incentives from the government (see also Qu et al., 2020). Thus, the government should regulate existing agricultural cooperatives to ensure they provide functions and services specific to farmer needs and educate farmers in this respect.

### 4.4 Implications for policy making

To facilitate the transition of apple production towards a more sustainable system, agricultural policies need to encourage farmers' adoption of new technologies and practices that deliver environmental and social benefits, and at the same time help farmers mitigate their risk associated with financial losses in production. It is only by simultaneously addressing socioeconomic and environmental factors in the policy mix that lock-ins to non-sustainable agricultural systems can be ended. The research here shows that developing policies which include policy levers relating to improving farmers' knowledge about orchard management by exploiting trust in information sources, building tighter and trusted networks involving farmers and buyers, boosting land consolidation, establishing platforms providing trustworthy information about production and sales, and co-producing new technologies/practices in line with farmers’ needs may break the lock-in situation. However, the lock-in to unsustainable agronomic practices can only be ended if other drivers within the system are considered. For example, in our research, good coordination and collaboration between different stakeholders may further develop niche markets for apple products based on sustainable cultivation practices. The economic viability of sustainable production is only possible if wholesalers and retailers endorse and support them.

The need to ensure the integration of stakeholder activities through the entirety of the supply chain can be demonstrated by the example of pest control. A few Chinese apple farmers use mechanical (e.g. sticky traps, insect-trap lights, and band and cardboard traps) and/or biological control methods (e.g. the release of pest predators and the use of insect pheromones) to control pests in apple orchards, while pest management evaluations and decisions may need greater inputs from agronomists and agricultural advisors, as well as policy levers to promote sustainable practices. These might include, for example, fiscal measures such as subsidies for sustainable technology adoption or taxation of unsustainable practice, and should benefit not only farmers but also other major stakeholders. Implementing effective traceability through the supply chain will increase stakeholder information sharing, which can be linked to consumer preferences for information delivery.

### 4.5 Research implications

This study departs from other studies that have employed ISM and MICMAC analysis to investigate sustainability-related issues. First, previous research has primarily focused on sustainable supply chain management from a focal company perspective, sometimes within specific sectors (e.g. see Agi and Nishant, 2017; Mathiyazhagan et al., 2013; Raut et al., 2017), but few have applied the analysis to agrifood sector (Gardas et al., 2019a). This research is centred on the apple production system. The approach adopted enables production system-specific recommendations to be made, increasing the development of pragmatic and actionable changes to reduce system lock-ins. The combination of structured surveys among farmers and in-depth interviews with different stakeholders could potentially reduce information bias and increase the validity of findings through the triangulation approach adopted. The feasibility of adopting the three-stage approach (i.e. conducting data collection, initial analysis, and further analysis using ISM and MICMAC methodologies) to investigate a particular industry within a specific cultural and policy context has been demonstrated, which can be applied to investigations of other industries in different cultural and policy contexts. However, this study has some limitations. This study only focuses on two apple production provinces, and differences might exist in apple production across all national production regions. Caution is thus needed when generalizing the findings to the national apple production system. Also, the specific results and recommendations cannot be generalized beyond the production system under consideration, as different agronomic and socio-economic conditions prevail within different agrifood systems.

## 5 Conclusion

The Chinese apple production system is associated with multiple environmental, economic and social problems, despite the government efforts focused on the implementation of technologies and practices designed to facilitate sustainable agriculture. The interactions of different elements through ISM analysis have further displayed the systemic dynamics and complexity, which hinders the adoption of sustainable production practices in China. Unsustainable practices have been locked-in to the system as a consequence of interacting socio-economic, agronomic and technological factors, and all should be targeted in the future policy mix designed to promote sustainable production. The three-stage approach employed in this study can accommodate both national and regional socio-economic, cultural, policy and agronomic contexts, which can be usefully applied to other agricultural supply chains in both the Global North and the Global South.

## Declaration of competing interest

The authors declare that they have no known competing financial interests or personal relationships that could have appeared to influence the work reported in this paper.

## Credit author statement

**Shan Jin:** Conceptualization, Methodology, Software, Data curation, Writing- Original draft preparation; **Wenjing Li:** Conceptualization, Funding acquisition, Data curation, Visualization, Writing- Reviewing and Editing; **Yiying Cao:** Reviewing and Editing; **Glyn Jones:** Reviewing and Editing; **Jing Chen:** Reviewing and Editing; **Zhenhong Li:** Reviewing and Editing; **Qian Chang:** Reviewing and Editing; **Guijun Yang:** Reviewing and Editing; **Lynn J. Frewer:** Supervision, Funding acquisition, Writing- Reviewing and Editing.
