# Supplementary material for: Identifying barriers to sustainable apple production: A stakeholder perspective
Source: J Environ Manage. 2022 Jan 15;302(Pt B):114082. doi: 10.1016/j.jenvman.2021.114082 (PMC8683745; doi:10.1016/j.jenvman.2021.114082)
Supplement: Multimedia component 1 [file mmc1.docx]

***Cover sheet for*** ***Supplementary material***

Shan Jin^1^, Wenjing Li^2,3,^*, Yiying Cao^4^, Glyn Jones^1,3^, Jing Chen^5^, Zhenhong Li^6,7^, Qian Chang^5,9^, Guijun Yang^8,9^ and Lynn J. Frewer^1^

*^1^ School of Natural and Environmental Sciences, Newcastle University, Newcastle upon Tyne, NE1 7RU, UK;*

*^2^ College of Economics and Management, Huazhong Agricultural University, Wuhan, 430070, China*

*^3^ Institute for Agri-Food Research and Innovation, FERA Sciences Ltd., National Agri-Food Innovation Campus, Sand Hutton, York YO41 1LZ, UK;*

*^4^ RSK ADAS Ltd, Spring Lodge, 172 Chester Road, Helsby, WA6 0AR, UK;*

*^5^ Institute of Agricultural Economics and Development, Chinese Academy of Agricultural Science, Beijing 100081, China;*

*^6^ College of Geological Engineering and Geomatics, Chang’an University, Xi’an 710054, China*

*^7^ School of Engineering, Newcastle University, Newcastle upon Tyne, NE1 7RU, UK*

*^8^ Key Laboratory of Quantitative Remote Sensing in Agriculture of Ministry of Agriculture and Rural Affairs, Beijing Research Center for Information Technology in Agriculture, Beijing 100097, China*

*^9^ National Engineering Research Center for Information Technology in Agriculture, Beijing 100097, China*

***Manuscript Title:*** *Identifying barriers to sustainable apple production: A stakeholder perspective*

*Number of pages: 13*

*Number of tables: 7*

*Number of figures: 0*

***Table of contents***

***Table S1****. Interviewee characteristics*

***Table S2.*** *Key environmental, economic and social problems in China’s apple production*

***Table S3.*** *Sources of information about agricultural chemical use reported by farmers in the survey*

***Table S4****. Drivers of farmers’ low adoption of new technologies and practices*

***Table S5.*** *List and SSIM of elements*

***Table S6.*** *Final reachability matrix*

***Table S7.*** *Level partitioning of elements*

***Appendix A:*** *Survey and interview data analyses*

***Table S1.*** ***Interviewee characteristics***

| **Location** | **Involved supply chain activities** | **Participant No.** | **Identity** | **Introduction** |
| --- | --- | --- | --- | --- |
| **Within apple  production areas** | **Apple production** | 1 | Farmer | The interviewee has about 9.7-hectare orchards in Shanxi province, of which 4 hectares leased from others. He has over 20 years' apple cultivation experience. |
|  |  | 2 | Farmer | The interviewee has about 7.3-hectare apple orchards in Shanxi province. He has over 30 years' apple cultivation experience. |
|  |  | 3 | Farmer | The interviewee has 8.5-hectare orchards in Shandong province and has over 20 years' apple cultivation experience. |
|  |  | 4 | Farmer | The interviewee has 12.5-hectare orchards with high-level standardization in Shandong province. He is a young farmer who came back from the urban area due to local government's support for young farmers. |
|  | **Apple production, warehousing and sales** | 5 | Chairman of an apple company; Chairman of a warehousing company;  Chairman of a fertilizer and pesticide manufacturing company; | This interviewee's company is one of the biggest apple companies in Shandong province. |
|  |  | 6 | Agricultural cooperative manager; Chairman of a warehousing company. | This interviewee's agricultural cooperative has 53 members in Shanxi province. It mainly provides farmers with warehousing service, online sales and cultivation guidance. |
|  |  | 7 | Chairman of an apple company;  Chairman of an agricultural cooperative; Chairman of a warehousing company. | This interviewee's agricultural cooperative has 122 members in Shanxi province. It mainly provides farmers with warehousing service, sales and cultivation guidance. He personally has a 20-hectare apple orchard with high-level mechanization. |
|  |  | 8 | Chairman of an apple company; Farmer. | The interviewee's company purchases apples from local farmers and sells apples via social media. He personally has 12.2-hectare orchards and has over 25 years' apple cultivation experience. |
|  |  | 9 | Chairman of an apple company | The interviewee's company has an 80.9-hectare orchard with high-level standardization and mechanization in Shandong province. It is one of the largest apple orchards in the local area. |
|  |  | 10 | Chairman of an apple company | The interviewee's company has a 267-hectare apple orchard with high-level standardization and mechanization in Shandong province. It is one of the largest orchards in the local area. |
|  |  | 11 | Chairman of an agricultural cooperative | The cooperative mainly provides farm chemicals and sales service for all members, who have a total of 809.4-hectare apple orchards. |
|  | **Regulations** | 12 | Village head;  Owner of an agricultural supply store;  Farmer. | The interviewee plays multiple roles in apple production in Shanxi province. Specifically, his family business in selling pesticides and fertilizers has lasted for over 30 years. |
|  |  | 13 | Staff of the Fruit Industry Bureau; Farmer. | The interviewee has worked in apple orchard technology and practice promotion for over 20 years in Shanxi province. Meanwhile, he personally has 2.4-hectare apple orchards. |
|  |  | 14 | Village head | The interviewee works for the local government in Shanxi province and is responsible for agricultural policy implementation. |
|  |  | 15 | Head of the County Fruit Industry Bureau | The interviewee is responsible for apple orchard technology and practice promotion in Shanxi province and has regular communication and collaboration with other stakeholders, such as farmers and apple companies. |
|  |  | 16 | Village head; Chairman of an agricultural cooperative. | The interviewee works closely with farmers and staffs from Fruit Industry Bureau in Shandong province. The agricultural cooperative, however, has just been established and could provide very limited services for local farmers. |
|  |  | 17 | Village head | The interviewee works for the local government in Shandong province and is responsible for agricultural policy implementation. |
|  |  | 18 | Head of the County Fruit Industry Bureau | The interviewee is responsible for apple orchard technology and practice promotion in Shandong province and has regular communication and collaboration with other stakeholders, such as farmers and apple companies. |
| **Outside apple  production areas** | **Apple purchase and logistics** | 19 | Independent broker | This interviewee has about 10 years' working experience in fruit procurement. |
|  |  | 20 | Manager of a fresh food supply chain company | The interviewee has over 10 years' apple procurement experience. |
|  |  | 21 | Manager of one of the biggest fruit retailers in China | The interviewee is responsible for apple procurement and sales. He has regular communication with big apple companies and wholesalers. |
|  |  | 22 | Manager of one of the biggest fruit wholesalers in China | The interviewee is responsible for apple procurement. He has regular communication with big apple companies, retailers and sub-wholesalers. |
|  |  | 23 | Manager of Walmart Food Safety Collaboration Centre | The interviewee is an expert in food safety and is familiar with fruit safety and quality control in procurement stage. |
|  | **Research into apple cultivation and markets** | 24 | Agronomist | This interviewee is a professor at an agricultural university, who has worked in the field of apple production for over 20 years. |
|  |  | 25 | Manager of a futures company | The interviewee is a professional in apple futures and is familiar with relevant transactions in China. |

***Table S2.*** *Key environmental, economic and social problems in China’s apple production*

| **ID** | **Description of themes** | **Evidence from the interviews** | **Evidence from the survey** |
| --- | --- | --- | --- |
| ***3.1.1 Environmental and health risks*** | | | |
| 3.1.1.1 | Synthetic agricultural chemicals were a source of environmental contamination in apple production. | *“In apple planting, there is pollution resulting from the use of synthetic pesticides, fertilizers, paper and plastic bags, and the reflective films*.” *(Participant 7, chairman of an apple company; an agricultural cooperative; and a warehousing company)* | 87.8% of survey respondents depended on synthetic pesticides application alone, with only 5.7% using mechanical control methods (e.g. sticky traps, insect-trap lights, and band and cardboard traps) and 5.3% using biological control methods (e.g. the release of pest predators and the use of insect pheromones). |
| 3.1.1.2 | Most farmers did not realise their ***lack of knowledge about orchard management***, resulting in environmental problems linked to *the* ***improper use of synthetic fertilizers and pesticides***. Spraying chemicals, negative health impacts might be experienced by bystanders and residents, despite farmers’ use of personal protective equipment. | *“Because the farmers have been planting for so many years, they feel that they are smarter and know how to plant apples. But they don’t actually know much about it. They ask an expert or other people for help only when some severe problems have emerged in production.” (Participant 13, a staff of County Fruit Industry Bureau and farmer)* | Farmers predominantly relied on their own experience in terms of chemical use, followed by advice from retailers of the chemicals they were applying (Table S3).  The majority of farmers perceived themselves to be knowledgeable about orchard management such as apple tree disease (89.39%) and pest management (90.61%). Despite so, 44.9% and 40.41% of farmers expressed the need to improve the disease and pest management practices, respectively. 62.86% of farmers expressed the need to improve the fertilizer practices. |
| 3.1.1.3 | Apple bagging was used for protecting apples from certain pests, promoting apple skin colouration and reducing blemishes. In ***the absence of effective disposal and recycling mechanisms***, the plastic bags, together with reflective films in orchards, represented a new environmental threat (**Fig. 2**). | *“We can instruct farmers to spray pesticides, but we can do nothing to help recover and recycle the reflective film and plastic bag waste in the field.” (Participant 7, chairman of an apple company; an agricultural cooperative; and a warehousing company)* | 97% of farmers surveyed reported adopting apple bagging, which used a total of 16.193 million bags in 2018, with plastic comprising 44% of these. |
| ***3.1.2 Yield instability*** | | | |
| 3.1.2.1 | Yield instability was a serious problem for farmers. ***Extreme climate events*** were considered to cause apple yields to plummet in certain areas, triggering price spikes across the country, demonstrating that the production system had low resilience to climate shocks. The changes in apple supply and demand then led to the instability of apple prices.  While some stakeholders mentioned the malicious hoarding of apples by big investors, the futures company manager suggested that this only exerted a very small influence on national price volatility. | *“The price of apples is low and unstable, and frost has this year has caused the yield loss.” (Participant 3, farmer)*  *“Last year, due to frost, all production areas had significant yield reduction, which caused the market to exceed supply. So, the apple price last year was very unstable.” (Participant 21, retailer)*  *“There must be people hoarding apples, but the overall quantity was small with limited impact on the national price. It was mainly due to the natural disaster last year and yield reduction.” (Participant 25, manager of a futures company)* | About 60% and 43% of farmers surveyed reported they suffered from yield loss caused by apple tree diseases and pests, respectively. The proportion of farmers suffering from pest-led yield loss was particularly higher in Shandong province (73%) than in Shanxi province (41%). |
| 3.1.2.2 | While cultivating dwarf varieties was considered to be able to mitigate negative impacts of climate conditions on production, it can be a heavy ***financial burden for apple producers***, in particular smallholder farmers, given the extra expenditure on saplings and supporting facilities and the income loss due to about 3 years’ growth to maturity. | *“The frost last year was particularly severe, and we don’t know if it will happen again next year, but the dwarf apple trees are easier to protect. However, if farmers shift to planting dwarf varieties, it will take three years to produce apples. They may be unwilling to do so as they can’t make money in the three years.” (Participant 7, chairman of an apple company; an agricultural cooperative; and a warehousing company)* | According to the survey, only 32% of farmers planted dwarf trees. |
| 3.1.2.3 | Despite the potential of agricultural insurance in mitigating financial risks caused by climatic extremes, farmers expressed low trust in insurance companies, resulting in the failure in promoting the insurance. | *“While insurance companies claim that yield loss caused by natural disasters will be compensated, they still reserve the right of final interpretation. Last year there was a frost and hail disaster, and in the end, the farmers did not receive compensation.” (Participant 12, village head; owner of an agricultural supply store; and farmer)* | About 24% of farmers surveyed reported they had the demand for apple production insurance. Only 7% of farmers in the survey bought agricultural insurance for their orchards. |
| ***3.1.3 Potential deterioration of apple quality*** | | | |
| 3.1.3.1 | Bigger, redder and blemish-free apples were often regarded as being of higher quality and could be sold at a higher price. Given the potential negative influence of pests, plant disease and climatic extremes, farmers were motivated to use farm chemicals, bagging and reflective films to ensure the “visual” quality of apples. The taste of apples appeared to have been negatively affected by the misuse of apple bagging and synthetic fertilizers. However, farmers often failed to realise their overuse of farm chemicals. | *“Good quality apples need to be blemish-free and red enough, which are easy to sell.” (Participant 1, farmer)*  *“In Xinjiang, we want all of them with diameters above 75 mm, and in Shaanxi, it is based on the same size. Apples in Shandong have been further classified according to their diameters, such as above 80 mm, 85 mm, 75 mm, and 70mm.” (Participant 21, retailer)*  *“Ordinary farmers always pursue yield by using more fertilizers and water more, and eventually produce apples with compromised taste.” (Participant 11, chairman of an agricultural cooperative)* | Less than 1% of farmers thought they overused pesticides in apple production in 2018. |
| ***3.1.4 Farmers’ uncertainty about accessing routes to market*** | | | |
| 3.1.4.1 | Smallholders’ sales depended on small independent brokers, apple companies or local warehousing companies for sales, but they normally had no regular contact or formal collaborations with wholesalers or retailers. In contrast, larger-scale farmers and apple companies tended to have more stable collaborations with wholesalers or retailers. | *“When the brokers come to this region, they would go to the warehousing companies, who then would ask if I would like to sell my apples at a certain price. I normally have to sell the apples as they cannot be well stored for a long time due to quality erosion.” (Participant 1, farmer)*  *“As I have a large orchard, I have signed contracts with large wholesalers and retailers, and the agreed prices will fluctuate with the market.” (Participant 9, chairman of an apple company)* | Only 32% of farmers surveyed were satisfied with their access to apple sales information. Two major barriers to obtaining sales information were limited knowledge about the channels providing sales information, and the huge amount of misinformation according to the survey results. |
| 3.1.4.2 | Farmers’ limited market access, when combined with their ***weak bargaining power*** relative to that of wholesalers and retailers, has led to uncertainty about how to initiate apple sales, causing negative impacts in particular on smallholders.  There has been ***lack of a trusted supplier-buyer relationship*** (between farmers and buyers) as the fruit supply chain companies, the wholesalers and the retailers primarily collaborated with apple companies or agricultural cooperatives. | *“There is an eagerness game between buyers and sellers, making farmers a disadvantaged group. When the apples are harvested, they must sell them. So, wholesalers or retailers often keep prices down. Farmers can't store many apples, which must be sold in a short time. So, the price would be low.” (Participant 25, manager of a futures company)*  *“Our cooperation objects are mainly divided into three categories, one is large growers in the apple production area, the others are the large local and city wholesalers.” (Participant 21, retailer)*  *“We don’t cooperate with farmers. They have unstable supply and sometimes may break the contract. We usually cooperate with local agricultural cooperatives or apple companies.” (Participant 22, wholesaler)* | Only 2% of farmers had fixed partnership with wholesalers or retailers. |

*Note*: **Text in bold** represents themes elicited via thematic analysis.

***Table S3.*** *Sources of information about agricultural chemical use reported by farmers in the survey*

| **Source** | **Total sample N=245** | **Shanxi**  **N=126** | **Shandong**  **N=119** |
| --- | --- | --- | --- |
| **Fertilizers** |  |  |  |
| Self-experience | 67% | 75% | 58% |
| Fertilizer retailers | 14% | 10% | 18% |
| Peer advice | 9% | 3% | 15% |
| Cooperatives' recommendations | 4% | 6% | 2% |
| Soil-testing | 4% | 5% | 3% |
| Agri-tech extension service from government | 3% | 2% | 3% |
| Advice from large-scale or model farmers | 1% | - | 2% |
| **Pesticides** |  |  |  |
| Self-experience | 65% | 71% | 58% |
| Pesticide retailers | 20% | 16% | 26% |
| Cooperatives' recommendations | 5% | 6% | 3% |
| Peer advice | 3% | 1% | 5% |
| Pesticide instructions | 3% | 3% | 3% |
| Agri-tech extension service from government | 2% | 1% | 3% |
| Apple companies' requirements | 1% | - | 1% |
| Advice from large-scale or model farmers | 1% | 2% | 1% |
| Packaged prescription and equipment | 1% | - | 1% |

***Table S4****. Drivers of farmers’ low adoption of new technologies and practices*

| **ID** | **Description of themes** | **Evidence from the interviews** | **Evidence from survey** |
| --- | --- | --- | --- |
| ***3.2.1 Land fragmentation and low standardization between orchards*** | | | |
| 3.2.1.1 | Land fragmentation limited farmers’ adoption of mechanization (e.g. irrigation and weeding), as it required sufficient orchard size and tree spacing. It was difficult to persuade farmers to standardize tree spacing as it potentially resulted in financial losses by reducing the number of trees planted. Consequentially, orchard management has become inefficient due to low standardization and mechanization, which had negative effects on apple yields and farmers’ income. | *“The problem with the promotion of mechanization is that you must be centralized and contiguous. However, the current land is highly fragmented, and some farmers are unwilling to transfer their lands.” (Participant 7, chairman of an apple company; an agricultural cooperative; and a warehousing company)*  *“It is very difficult to promote new technologies or varieties here. The trees have been planted for so many years. Farmers are unwilling to cut them down and replace them with new ones. Cutting down the trees represents income loss.” (Participant 6, manager of an agricultural cooperative and chairman of a warehousing company)*  *“If you want to grow enough apples with a diameter of 80 mm, you need to have larger-scale orchards. Although we currently have over 100 cooperative members, their orchards are too scattered, and it’s difficult to manage. The planting scale is also uneven. Some orchards are only a few mu, and some are more than ten mu.” (Participant 7, chairman of an apple company; an agricultural cooperative; and a warehousing company)* | According to the survey data, the number of orchard blocks for each farmer varies from 1 to 30, and 76% of the farmers have a farm size of less than 1 hectare. In the same region, different orchards had different tree spacing regimes and management practices (e.g. the use of agricultural chemicals, or blossom and fruit thinning). |
| ***3.2.2 Lack of long-term apple cultivation plans*** | | | |
| 3.2.2.1 | ***The ageing farming workforce and young people’s rural-to-urban migration*** was a frequently mentioned problem and acted as a barrier to the long-term plan for improving apple production. Despite the government’s offer of training courses for young people, few ultimately continued working in apple production. | *“The ageing here is very serious. The young people have moved to cities. Those who stay in the countryside are mainly in their 60s. They couldn't do labour intensive work and tend to not carefully and scientifically manage their orchards.” (Participant 17)*  *“We had training courses every year to attract young people, and they were always fully booked, while very few young people ultimately stayed to grow apples. It is because of financial limitations.” (Participant 15, head of County Fruit Industry Bureau)* | The survey data showed that 36% of farmers planned to decrease the area of, or phase out, apple cultivation in the future. These farmers could be less likely to adopt novel technologies or practices in orchard management. Only 9% of farmers planned to enlarge the size of their orchards, which would act as a barrier to farm mechanization. |
| ***3.2.3 Financial difficulties*** | | | |
| 3.2.3.1 | Farmers had limited budgets for investment in technologies. As such, farmers’ access to financial support might significantly affect their investment in apple production. For those who planned to expand their orchard sizes, the difficulty of getting loans from financial institutions could act as a barrier to shift to more environmentally friendly technologies and practices. | *“I don't know about other places. In this place, apple producers cannot raise capital, and it is impossible for the bank to lend you a loan. We don’t have financing channels but borrowing money via private networks.” (Participant 10, chairman of an apple company)* | There were about 43% and 40% of farmers reporting financial difficulty in purchasing precision fertilizer and pesticide equipment, respectively. |
| ***3.2.4 Low awareness and interests of sustainable orchard management*** | | | |
| 3.2.4.1 | Overall, farmers had low awareness of and interests in sustainability issues associated with apple production. Other stakeholders such as the wholesaler and retailer mentioned the importance of environmental conservation. However, they did not include the environmental impacts of production as a criterion for supplier selection, representing ***lack of buyer pressure to drive sustainable production***. | *“There are some new technologies, but I just care about saving labour and money, such as using the integrated water and fertilizer equipment, the main labour costs are now too high.” (Participant 2, farmer)*  *“In fact, farmers will not consider environmental pollution, because most are smallholders, and are not aware of it in this area. We can't require them to reduce pesticide use or recover bags as there is no legal regulation. Therefore, they always overuse pesticides to ensure production and their income.” (Participant 15, head of County Fruit Industry Bureau)* | There were 41% of farmers claiming the need to reduce negative impacts on the environment, of whom only 10% viewed reducing negative impacts on the environment as their priority. |
| ***3.2.5 Limited access to trustworthy information*** | | | |
| 3.2.5.1 | Training is an effective way for farmers to improve their knowledge and skills in sustainable farming practices. Some training farmers attended was provided by chemical manufacturers. | *“There will be training every year, such as those who sell chemical fertilizers and pesticides, and technicians also give lectures.” (Participant 6, manager of an agricultural cooperative and chairman of a warehousing company)* | There were 28% of farmers reporting difficulties in accessing training in apple cultivation. |
|  | No regular contact had been established between farmers and big retailers or wholesalers. Farmers depended on small independent brokers, apple companies or warehousing companies for sales, but with a low trust, which could act as a barrier to information exchange, for example, about wholesalers’ or retailers’ procurement. | *“We directly sell apples to the small brokers as soon as possible. If some farmers don’t want to sell right now, they store apples in warehousing companies and wait for other buyers.” (Participant 15, head of County Fruit Industry Bureau)*  *“Our domestic trust is very low, especially between apple purchasers and suppliers. The current apple purchases are sometimes messy.” (Participant 20, manager of a fresh food supply chain company)*  *“The ideal situation would be selling apples to certain buyers every year. However, the reality is we never know whether the buyers will come next year. They normally won’t come back.” (Participant 13, a staff of the County Fruit Industry Bureau and farmer)* | Only 2% of farmers had fixed partnership with wholesalers or retailers. |
| ***3.2.6 Limited development and promotion of novel technologies and practices*** | | | |
| 3.2.6.1 | Difficulties and failures associated with novel technology/practice promotion in apple production areas reflected ***low adoption willingness among farmers***. A lack of technologies that help with the proper use of pesticides in apple cultivation was identified. | *“We go out to visit large companies every year, all of which are of high level. My 50-mu land now uses integrated water and fertilizer equipment, saving a lot of labour and more efficient. You can see the effect immediately after three days.” (Participant 13, a staff of County Fruit Industry Bureau and farmer)* | Soil testing and fertilizer recommendation services were promoted in both Shanxi and Shandong provinces. However, the survey indicated that only 18% of farmers had used these services. |
| ***3.2.7 High levels of risk perceptions linked to financial loss*** | | | |
| 3.2.7.1 | Farmers were concerned about yield loss and quality decline due to the significant impact on income. Therefore, they actively used chemicals to protect apple trees from pests and diseases, a behaviour which could be difficult to change. Farmers might perceive that reducing chemical inputs increases the risk of yield loss or has negative effects on the visual quality of the apples. | *“Most of them here are smallholders. It is not “the possible overuse of pesticides”, it is “100% overuse of pesticides”. Given the low costs, they always spray more at one time, mainly because they’re concerned about yield loss due to the insufficient quantity of pesticide use.” (Participant 15, head of County Fruit Industry Bureau)* | 70% of the surveyed farmers reported apple sales accounted for over half their household income. About 60% and 43% of farmers surveyed reported they suffered from yield loss caused by apple tree diseases and pests, respectively.  With respect to farmers’ risk attitudes, 84% of farmers were risk-averse, 13% of farmers were risk tolerant, and 3% were neutral. |
| ***3.2.8 Ineffective agricultural policies*** | | | |
| 3.2.8.1 | The Chinese government provided subsidies for purchasing organic fertilizers, but farmers were still more dependent on synthetic fertilizers.  Existing agricultural policies did not include some potentially problematic areas of production. For example, policies targeting the reduction of pesticide use and other environmental pollutants (e.g. plastic bags) have not been enacted. | *“There were subsidies for buying organic fertilizers before, but the main reliance is still synthetic fertilizers, which is relatively stable. The fertility of organic fertilizers is still not stable sometimes, and often slowly comes into effect.” (Participant 16, village head and chairman of an agricultural cooperative)*  *“You can't require them (farmers) to reduce pesticide use or recover bags as there is no legal regulation. It can only depend on self-discipline, which is very hard to realise.” (Participant 15, head of County Fruit Industry Bureau)* | The use of organic fertilizers accounted for less than 31% of the total fertilization applications, showing still the dominance of synthetic fertilizers. |
| 3.2.8.2 | Many agricultural cooperatives were established in different apple production regions after the Law of the People’s Republic of China on Farmers’ Professional Cooperatives was implemented. They were intended to benefit smallholder farmers by providing different services, such as technical training, facilitating apple sales and enabling collective purchases of agricultural inputs. In fact, a few cooperatives in Shanxi province were founded by apple companies, pesticide or fertilizer retailers or warehousing companies. Some interview participants questioned the value of the functions or services agricultural cooperatives provided for farmers and considered these cooperatives primarily as a means of obtaining financial incentives from the government. | *“At present, the cooperative provides little help for farmers. They just sometimes sell fertilizers to farmers.” (Participant 4, farmer)*  *“In fact, the agricultural cooperatives here haven't really played a role in helping farmers sell apples. Even if there are cooperative helping with that, it would be an extremely small number.” (Participant 15, head of County Fruit Industry Bureau)*  *“The agricultural cooperatives here are more like doing business, with a few partners and no farmers in the end. Initially, cooperatives were established, mainly intended for getting some national demonstration projects, but the standard gradually became stricter. So, some cooperatives became shell corporations.” (Participant 13, a staff of County Fruit Industry Bureau and farmer)* | The survey showed that 39% of farmers (*N* = 96) joined agricultural cooperatives. The main reason for farmers not joining agricultural cooperatives was the limited functions of agriculture cooperatives, accounting for 45% of the non-members. |

*Note*: **Text in bold** represents themes elicited via thematic analysis.

***Appendix B:*** *Details of ISM*

The ISM was deployed in the following ***three steps***:

***Step 1: Development of Structural Self-Interaction Matrix (SSIM)***

The SSIM of elements was coded following the criteria below (*where four symbols indicate the direction of influence between different elements: “V” denotes that element “i” affects “j”; “A” denotes element “j” affects “i”; “X” denotes element “i” affects “j” and element “j” affects “i”; and “O” denotes elements “i” and “j” are independent of each other*) and results were presented in ***Table S5***.

***Table S5.*** *List and SSIM of elements*

| Elements | E21 | E20 | E19 | E18 | E17 | | E16 | | E15 | | | E14 | E13 | E12 | E11 | E10 | E9 | E8 | E7 | E6 | E5 | E4 | E3 | E2 | E1 |  |
| --- | --- | --- | --- | --- | --- | --- | --- | --- | --- | --- | --- | --- | --- | --- | --- | --- | --- | --- | --- | --- | --- | --- | --- | --- | --- | --- |
| E1 | A | A | O | O | O | | A | | O | | | O | O | O | O | O | O | O | O | A | A | O | O | O | X |  |
| E2 | O | A | V | O | O | | O | | V | | | O | V | A | O | O | O | A | A | X | X | V | O | X |  |  |
| E3 | O | A | V | O | O | | O | | V | | | O | O | A | O | V | O | A | A | X | X | V | X |  |  |  |
| E4 | A | O | O | O | A | | O | | V | | | O | O | O | A | A | O | O | O | O | O | X |  |  |  |  |
| E5 | A | A | A | A | A | | A | | O | | | O | O | O | O | O | A | A | A | A | X |  |  |  |  |  |
| E6 | A | A | A | A | A | | A | | O | | | O | O | O | O | O | A | A | A | X |  |  |  |  |  |  |
| E7 | O | V | V | A | A | | A | | O | | | O | O | V | O | O | O | O | X |  |  |  |  |  |  |  |
| E8 | O | O | V | A | A | | O | | O | | | O | O | O | O | O | O | X |  |  |  |  |  |  |  |  |
| E9 | A | V | O | O | O | | V | | O | | | O | O | O | O | O | X |  |  |  |  |  |  |  |  |  |
| E10 | O | O | O | O | A | | O | | O | | | O | O | O | A | X |  |  |  |  |  |  |  |  |  |  |
| E11 | A | O | O | O | V | | O | | O | | | O | O | O | X |  |  |  |  |  |  |  |  |  |  |  |
| E12 | A | V | O | V | O | | O | | O | | | O | A | X |  |  |  |  |  |  |  |  |  |  |  |  |
| E13 | O | V | O | O | O | | O | | O | | | A | X |  |  |  |  |  |  |  |  |  |  |  |  |  |
| E14 | A | O | O | O | O | | O | | O | | | X |  |  |  |  |  |  |  |  |  |  |  |  |  |  |
| E15 | A | V | V | O | O | | O | | X | | |  |  |  |  |  |  |  |  |  |  |  |  |  |  |  |
| E16 | A | V | A | O | A | | X | |  | | |  |  |  |  |  |  |  |  |  |  |  |  |  |  |  |
| E17 | A | V | V | O | X | |  | |  | | |  |  |  |  |  |  |  |  |  |  |  |  |  |  |  |
| E18 | A | V | V | X |  | |  | |  | | |  |  |  |  |  |  |  |  |  |  |  |  |  |  |  |
| E19 | A | V | X |  |  | |  | | |  | |  |  |  |  |  |  |  |  |  |  |  |  |  |  |  |
| E20 | A | X |  |  | |  | |  | | |  |  |  |  |  |  |  |  |  |  |  |  |  |  |  |  |
| E21 | X |  |  |  | |  | | |  | |  |  |  |  |  |  |  |  |  |  |  |  |  |  |  | |

***Step2: Building reachability matrix***

The initial reachability matrix was formulated from SSIM following the rules:

- If the (i, j) in the SSIM contains “V”, the (i, j) entry in the reachability matrix is 1 and the corresponding (j, i) entry is 0;
- If the (i, j) in the SSIM contains “A”, the (i, j) entry in the reachability matrix is 0 and the corresponding (j, i) entry is 1;
- If the (i, j) in the SSIM contains “X”, the (i, j) entry in the reachability matrix is 1 and the corresponding (j, i) entry is also 1; and
- If the (i, j) in the SSIM contains “O”, the (i, j) entry in the reachability matrix is 0 and the corresponding (j, i) entry is also 0.

The resulting matrix was then checked for transitivity, which is a basic assumption stating that if a variable A affects B and B affects C, then A necessarily affects C. After considering the transitivity rule, 0 in some cells was transformed into 1, labelled with a “*”. The results are presented in the final reachability matrix (***Table S6*)**, which also included the driving and dependence powers of each element. The driving power denotes the total number of elements (including itself), which a specific element can affect, whereas the dependence power denotes the total number of elements (including itself), which can affect a specific element. These driving and dependence powers were used to conduct the MICMAC analysis.

***Table S6.*** *Final reachability matrix*

| Elements | E1 | E2 | E3 | E4 | E5 | E6 | E7 | E8 | E9 | E10 | E11 | E12 | E13 | E14 | E15 | E16 | E17 | E18 | E19 | E20 | E21 | Driving power |
| --- | --- | --- | --- | --- | --- | --- | --- | --- | --- | --- | --- | --- | --- | --- | --- | --- | --- | --- | --- | --- | --- | --- |
| E1 | 1 | 0 | 0 | 0 | 0 | 0 | 0 | 0 | 0 | 0 | 0 | 0 | 0 | 0 | 0 | 0 | 0 | 0 | 0 | 0 | 0 | 1 |
| E2 | 1* | 1 | 1* | 1 | 1 | 1 | 0 | 0 | 0 | 0 | 0 | 1* | 1 | 0 | 1 | 1* | 0 | 0 | 1 | 1* | 0 | 12 |
| E3 | 1* | 1* | 1 | 1 | 1 | 1 | 0 | 0 | 0 | 1 | 0 | 0 | 0 | 0 | 1 | 1* | 0 | 0 | 1 | 1* | 0 | 11 |
| E4 | 0 | 0 | 0 | 1 | 0 | 0 | 0 | 0 | 0 | 0 | 0 | 0 | 0 | 0 | 1 | 0 | 0 | 0 | 1* | 1* | 0 | 4 |
| E5 | 1 | 1 | 1 | 1* | 1 | 1* | 0 | 0 | 0 | 1* | 0 | 0 | 1* | 0 | 1* | 0 | 0 | 0 | 1* | 0 | 0 | 10 |
| E6 | 1 | 1 | 1 | 1* | 1 | 1 | 0 | 0 | 0 | 1* | 0 | 0 | 1* | 0 | 1* | 0 | 0 | 0 | 1* | 0 | 0 | 10 |
| E7 | 1* | 1 | 1 | 1* | 1 | 1 | 1 | 0 | 0 | 1* | 0 | 1 | 1* | 0 | 1* | 1* | 0 | 1* | 1 | 1 | 0 | 15 |
| E8 | 1* | 1 | 1 | 1* | 1 | 1 | 0 | 1 | 0 | 1* | 0 | 0 | 1* | 0 | 1* | 1* | 0 | 0 | 1 | 1* | 0 | 13 |
| E9 | 1* | 1* | 1* | 0 | 1 | 1 | 1* | 0 | 1 | 0 | 0 | 0 | 0 | 0 | 0 | 1 | 0 | 0 | 0 | 1 | 0 | 9 |
| E10 | 0 | 0 | 0 | 1 | 0 | 0 | 0 | 0 | 0 | 1 | 0 | 0 | 0 | 0 | 1* | 0 | 0 | 0 | 0 | 0 | 0 | 3 |
| E11 | 0 | 0 | 0 | 1 | 1* | 1* | 1* | 1* | 0 | 1 | 1 | 0 | 0 | 0 | 1* | 1* | 1 | 0 | 1* | 1* | 0 | 12 |
| E12 | 1* | 1 | 1 | 1* | 1* | 1* | 1* | 1* | 0 | 1* | 0 | 1 | 1* | 0 | 1* | 0 | 0 | 1 | 1* | 1 | 0 | 15 |
| E13 | 1* | 1* | 1* | 0 | 1* | 1* | 0 | 0 | 0 | 0 | 0 | 1 | 1 | 0 | 0 | 0 | 0 | 1* | 0 | 1 | 0 | 9 |
| E14 | 0 | 0 | 0 | 0 | 0 | 0 | 0 | 0 | 0 | 0 | 0 | 1* | 1 | 1 | 0 | 0 | 0 | 0 | 0 | 1* | 0 | 4 |
| E15 | 1* | 1* | 1* | 0 | 1* | 1* | 0 | 0 | 0 | 0 | 0 | 0 | 0 | 0 | 1 | 1* | 0 | 0 | 1 | 1 | 0 | 9 |
| E16 | 1 | 1* | 1* | 0 | 1 | 1 | 1 | 0 | 0 | 0 | 0 | 1* | 0 | 0 | 0 | 1 | 0 | 0 | 1* | 1 | 0 | 10 |
| E17 | 1* | 1* | 1* | 1 | 1 | 1 | 1 | 1 | 0 | 1 | 0 | 1* | 0 | 0 | 1* | 1 | 1 | 0 | 1 | 1 | 0 | 15 |
| E18 | 1* | 1* | 1* | 0 | 1 | 1 | 1 | 1 | 0 | 0 | 0 | 1* | 0 | 0 | 0 | 1* | 0 | 1 | 1 | 1 | 0 | 12 |
| E19 | 1* | 1* | 1* | 0 | 1 | 1 | 1* | 0 | 0 | 0 | 0 | 0 | 0 | 0 | 0 | 1 | 0 | 0 | 1 | 1 | 0 | 9 |
| E20 | 1 | 1 | 1 | 1* | 1 | 1 | 0 | 0 | 0 | 1* | 0 | 0 | 1* | 0 | 1* | 0 | 0 | 0 | 1* | 1 | 0 | 11 |
| E21 | 1 | 1* | 1* | 1 | 1 | 1 | 1* | 1* | 1 | 1* | 1 | 1 | 1* | 1 | 1 | 1 | 1 | 1 | 1 | 1 | 1 | 21 |
| Dependence power | 17 | 16 | 16 | 13 | 17 | 17 | 9 | 6 | 2 | 11 | 2 | 9 | 10 | 2 | 14 | 12 | 3 | 5 | 16 | 17 | 1 |  |

*Note*: * refers to the factor that was transformed from 0 into 1.

***Step 3: Levels partitioning and formulation of ISM-based model***

Based on ***Table S5***, each element was accompanied by its reachable, antecedent elements and their overlaps as the intersection set. Elements with identical reachability and intersection sets were assigned at level 1. After the level 1 element was eliminated from the table, the remaining elements with identical reachability and intersection sets were then recorded as level 2. The iterative process was conducted until all the elements were categorized into different levels. In this study, 21 elements were classified into 11 levels. The reachability, antecedent and intersection sets, together with the calculated level for each element, are shown in ***Table S7***. Based on the results of levels partitioning, the hierarchical structural model was established in ***Fig. 3***, in which the most independent elements are E21 “Ineffective agricultural policies” and E11 “Lack of trusted supplier-buyer relationship”, with E21 having the highest influence on the other elements within this system and currently being impacted by none of these identified elements.

***Table S7*.** Level partitioning of elements

| **Element** | **Reachability set** | **Antecedent set** | **Intersection set** | **Level** |
| --- | --- | --- | --- | --- |
| 1 | 1 | 1,2,3,5,6,7,8,9,12,13,15,16,17,18,19,20,21 | 1 | 1 |
| 2 | 1,2,3,4,5,6,12,13,15,16,19,20 | 2,3,5,6,7,8,9,12,13,15,16,17,18,19,20,21 | 2,3,5,6,12,13,15,16,19,20 | 3 |
| 3 | 1,2,3,4,5,6,10,15,16,19,20 | 2,3,5,6,7,8,9,12,13,15,16,17,18,19,20,21 | 2,3,5,6,15,16,19,20 | 5 |
| 4 | 4,15,19,20 | 2,3,4,5,6,7,8,10,11,12,20,21 | 4,20 | 3 |
| 5 | 1,2,3,4,5,6,10,13,15,19 | 2,3,5,6,7,8,9,11,12,13,15,16,17,18,19,20,21 | 2,3,5,6,13,15,19 | 5 |
| 6 | 1,2,3,4,5,6,10,13,15,19 | 2,3,5,6,7,8,9,11,12,13,15,16,17,18,19,20,21 | 2,3,5,6,13,15,19 | 5 |
| 7 | 1,2,3,4,5,6,7,10,12,13,15,16,18,19,20 | 7,9,11,12,16,17,18,19,21 | 7,12,16,18,19 | 7 |
| 8 | 1,2,3,4,5,6,8,10,13,15,16,19,20 | 8,11,12,17,18,21 | 8 | 8 |
| 9 | 1,2,3,5,6,7,9,16,20 | 9,21 | 9 | 8 |
| 10 | 4,10,15 | 3,5,6,7,8,10,11,12,17,20,21 | 10 | 4 |
| 11 | 4,5,6,7,8,10,11,15,16,17,19,20 | 11,21 | 11 | 10 |
| 12 | 1,2,3,4,5,6,7,8,10,12,13,15,18,19,20 | 2,7,12,13,14,16,17,18,21 | 2,7,12,13,18 | 7 |
| 13 | 1,2,3,5,6,12,13,18,20 | 2,5,6,7,8,12,13,14,20,21 | 2,5,6,12,13,20 | 7 |
| 14 | 12,13,14,20 | 14,21 | 14 | 8 |
| 15 | 1,2,3,5,6,15,16,19,20 | 2,3,4,5,6,7,8,10,11,12,15,17,20,21 | 2,3,5,6,15,20 | 3 |
| 16 | 1,2,3,5,6,7,12,16,19,20 | 2,3,7,8,9,11,15,16,17,18,19,21 | 2,3,7,16,19 | 7 |
| 17 | 1,2,3,4,5,6,7,8,10,12,15,16,17,19,20 | 11,17,21 | 17 | 9 |
| 18 | 1,2,3,5,6,7,8,12,16,18,19,20 | 7,12,13,18,21 | 7,12,18 | 9 |
| 19 | 1,2,3,5,6,7,16,19,20 | 2,3,4,5,6,7,8,11,12,15,16,17,18,19,20,21 | 2,3,5,6,7,16,19,20 | 2 |
| 20 | 1,2,3,4,5,6,10,13,15,19,20 | 2,3,4,7,8,9,11,12,13,14,15,16,17,18,19,20,21 | 2,3,4,13,15,19,20 | 6 |
| 21 | 1,2,3,4,5,6,7,8,9,10,11,12,13,14,15,16,17,18,19,20,21 | 21 | 21 | 11 |
